# Colesterol Não Controlado em Indivíduos com Hipercolesterolemia Grave Acompanhados em um Programa de Avaliação da Saúde no Brasil

**DOI:** 10.36660/abc.20240116

**Published:** 2024-10-30

**Authors:** Raul D. Santos, Nea Miwa Kashiwagi, Fernando Yue Cesena, Silvia Regina Lamas Assis, Josué Nieri, Carlos Andre Minanni, Marcelo Franken, Otavio Berwanger

**Affiliations:** 1 Hospital Israelita Albert Einstein São Paulo SP Brasil Hospital Israelita Albert Einstein, São Paulo, SP – Brasil; 2 Hospital das Clínicas Faculdade de Medicina Universidade de São Paulo São Paulo SP Brasil Instituto do Coração do Hospital das Clínicas da Faculdade de Medicina da Universidade de São Paulo, São Paulo, SP – Brasil; 3 Instituto Dante Pazzanese de Cardiologia São Paulo SP Brasil Instituto Dante Pazzanese de Cardiologia, São Paulo, SP – Brasil; 4 Cenocor Guarulhos SP Brasil Cenocor, Guarulhos, SP – Brasil; 5 George Institute for Global Health UK Londres Reino Unido George Institute for Global Health UK, Londres – Reino Unido

**Keywords:** Colesterol, Fatores de Risco de Doenças Cardíacas, Hipercolesterolemia

## Abstract

**Fundamento:**

Indivíduos com hipercolesterolemia grave (HG) são considerados sob alto risco de desenvolverem aterosclerose e devem ser intensivamente tratados com medicamentos hipolipemiantes, visando uma redução nos níveis de LDL-Colesterol ≥50% e uma meta <70 mg/dL.

**Objetivos:**

Este estudo teve como objetivo avaliar o controle dos níveis de colesterol em indivíduos com HG (LDL-C ≥ 190 mg/dL ou 160-189 mg/dL usando medicamentos hipolipemiantes) acompanhados em um programa de avaliação da saúde.

**Métodos:**

Foram avaliados 55 000 indivíduos, dos quais 2214 (4%) apresentavam HG, e 1016 (45,8%) foram submetidos a avaliações repetidas. O alcance das metas de LDL-C foi o desfecho primário do estudo. Um valor de p<0,05 foi considerado significativo.

**Resultados:**

A idade média (±DP) foi 44,9±8,8 anos; 84,2% dos participantes eram do sexo masculino, e 0,5% relataram infarto do miocárdio prévio. A concentração média de LDL-C foi 203,0±22,0 mg/dL, e apesar de 62,5% dos pacientes terem referido dislipidemia, somente 19% estavam usando drogas hipolipemiantes (5,9% nos casos de LDL-C ≥ 190 mg/dL). Durante um seguimento de 4,1±2,8 anos, o uso de medicamentos hipolipemiantes aumentou de 18,1% para 48,4% (p<0,00001); de 5,9% para 45,4% naqueles com LDL-C ≥ 190 mg/dL (p< 0,00001), embora 31% dos casos com LDL-C 160-189 mg/dL terem interrompido o uso desses fármacos. No geral, observou-se uma redução média de 26,7% nos níveis de LDL-C (p<0,0001). Reduções ≥50% no LDL-C foram alcançadas por 19,2%, 19,1%, e 19,7 % de todos os indivíduos, e naqueles com LDL-C > 190 mg/dL e 160-189 mg/dL, respectivamente. Somente 3,1% atingiram concentrações de LDL-C < 70 mg/dL (2,7% naqueles com LDL-C ≥ 190 e 5,3% naqueles com 160-189 mg/dL).

**Conclusões:**

Uma séria lacuna foi encontrada entre as recomendações de tratamento e a realidade em indivíduos com elevado risco aterosclerótico por HG.

## Graphical abstract

**Figura Central:**
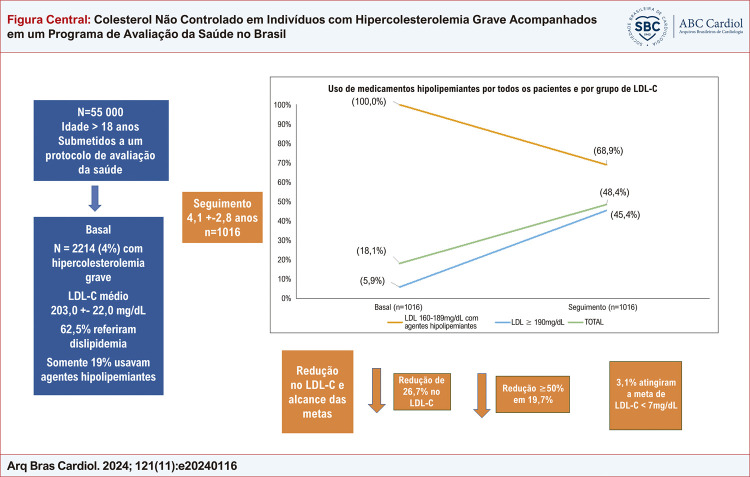
Colesterol Não Controlado em Indivíduos com Hipercolesterolemia Grave Acompanhados em um Programa de Avaliação da Saúde no Brasil

Ausência de controle dos níveis de colesterol na hipercolesterolemia grave.

## Introdução

A doença cardiovascular aterosclerótica (DCVA) é a principal causa de morte no Brasil.^1^ O LDL-colesterol (LDL-C) plasmático é um fator de risco causal para DCVA.^2-4^ A determinação das concentrações do LDL-C é, portanto, uma ferramenta essencial para estimar o risco de DCVA e implementar terapias preventivas. Adultos com hipercolesterolemia grave (HG), definida como LDL-C ≥ 190 mg/dL, são classificados como indivíduos com alto risco de DCVA independentemente de outras condições de risco, e as diretrizes recomendam terapia com agentes hipolipemiantes e mudanças no estilo de vida para prevenir o início de DCVA. Para esses indivíduos, diretrizes dos EUA, Brasil e Europa recomendam uma redução nos níveis de LDL-C de pelo menos 50%, com metas de LDL-C < 70 mg/dL nas duas últimas diretrizes.^2-4^ Além disso, concentrações persistentes de LDL-C entre 160 e 189 mg/dL estão associadas a um risco elevado de DCVA ao longo da vida, são consideradas potenciadores do risco, e favorecem o início da terapia com estatina.^2,5^

Programas de avaliação ou *checkups* de saúde têm como objetivo identificar fatores de risco para DCVA e encaminhar os indivíduos à assistência médica adequada quando necessária.^6^ Nós detectamos previamente uma importante lacuna na percepção do risco para DCVA, no manejo dos níveis de colesterol, e consciência sobre a hipercolesterolemia familiar em indivíduos com HG submetidos à avaliação de rotina.^7^ Esses achados complementaram outra observação mostrando uma percepção inadequada do risco global de DCVA por indivíduos em risco que possam ter consequências deletérias para o controle de fatores de risco e prevenção de DCVA.^8^ A falta de controle adequado dos fatores de risco ou estado da doença, e a ausência de adesão às recomendações após os programas de *checkup* é um desafio para os programas de medicina preventiva^6^ e precisa ser abordada. O objetivo deste estudo foi gerar evidência de mundo real (EMR) sobre a eficácia do controle do LDL-C de acordo com diretrizes recentes em indivíduos com HG submetidos a avaliações repetidas em um programa de avaliação de saúde de rotina. O desfecho primário foi o alcance das metas de LDL-C recomendadas para esses indivíduos em alto risco (redução ≥50% e/ou LDL-C <70 mg/dL).

## Métodos

Este estudo consiste em uma avaliação retrospectiva de dados coletados prospectivamente de adultos submetidos a um protocolo de avaliação de rotina do estado de saúde em um ambulatório de um hospital terciário em São Paulo, Brasil. A maioria dos participantes trabalharam em empresas que ofereciam avaliações rotineiras de saúde a seus empregados. Este estudo foi aprovado pelo comitê de ética (CAAE número 25772619.0000.00.0071), e se obteve dispensa do termo de consentimento. Este estudo foi financiado pelo laboratório Amgen Brasil, e a fonte financiadora não participou da coleta, análise, e interpretação dos dados, conclusões do estudo, ou elaboração do manuscrito.

Os critérios de inclusão foram a) indivíduos com idade ≥ 18 anos com HG definida como LDL-C ≥190mg/dL ou b) LDL-C entre 160 e 189 mg/dL naqueles usando medicamentos hipolipemiantes, acompanhados no ambulatório entre outubro de 2004 e novembro de 2019. O protocolo de avaliação de saúde consistiu em exame clínico (uso de questionários para DCVA prévia e seus fatores de risco, pressão arterial, diabetes, dislipidemia, e medicamentos para seus controles), e exames laboratoriais em jejum como descrito anteriormente.^7,9^ Um relatório de avaliação foi enviado ou para os participantes ou para o médico ocupacional da empresa quando disponível, e os participantes foram convidados para uma segunda entrevista com médicos examinadores para discutir os resultados do teste. Os participantes foram aconselhados e encaminhados para um especialista em caso de resultados anormais. Normalmente, nenhum medicamento foi prescrito pelo médico que realizava o *check-up*. Os seguintes parâmetros foram extraídos do prontuário médico eletrônico: idade, sexo, índice de massa corporal, presença de fatores de risco para DCVA tais como hipertensão arterial, diabetes, dislipidemia, tabagismo atual, história familiar de doença arterial coronariana (DAC) precoce (<55 em mulheres e < 65 anos em homens), síndrome metabólica definida de acordo com a Federação Internacional do Diabetes,^4^ e DAC definida como infarto do miocárdio, angina ou revascularização. Os dados sobre perfis lipídicos em jejum (colesterol total, LDL-C e triglicerídeos) também foram coletados.

Dos indivíduos avaliados em mais de uma ocasião, foram coletadas informações sobre o uso ou não de drogas hipolipemiantes e o alcance de metas de LDL-C de acordo com recomendações, isto é, porcentagem de indivíduos com LDL-C <100 mg/dL, <70 mg/dL, e <50 mg/dL durante o seguimento.^3,4^ Além disso, foram determinas as mudanças médias de porcentagem no LDL-C desde a primeira avaliação e a porcentagem dos indivíduos que se mantiveram uma redução ≥ 50% nos níveis de LDL-C a partir do basal, como recomendado por diretrizes^2-4^ para indivíduos em alto risco para DCVA. Para pacientes com mais de uma avaliação, dados da última visita foram considerados para análise.

### Análise estatística

A normalidade dos dados foi testada pelo teste de Kolmogorov-Smirnov; dados contínuos com distribuição gaussiana são apresentados como média ± desvio padrão (DP), dados não gaussianos são apresentados como mediana (intervalo interquartil), e variáveis categóricas são apresentadas como frequências relativas e absolutas.

Para a análise, os indivíduos foram primeiramente categorizados e agrupados de acordo com os valores de LDL-C durante o acompanhamento como > 100, 100-70, <70-50, e <50. Análise descritiva foi usada, e os grupos foram comprados pelo teste t de Student pareado. Os dados categóricos foram comparados usando o teste do qui-quadrado ou o teste de exato de Fisher. Modelos de regressão logística *stepwise* univariada e multivariada foram usados para avaliar a associação das variáveis clínicas de interesse com terapia com medicamentos hipolipemiantes na última avaliação clínica durante o seguimento. Um p-valor bicaudal < 0,05 foi considerado significativo. Todas as análises foram realizadas usando o software R versão 4.1.1 (R Foundation).

## Resultados

**Figura 1:**
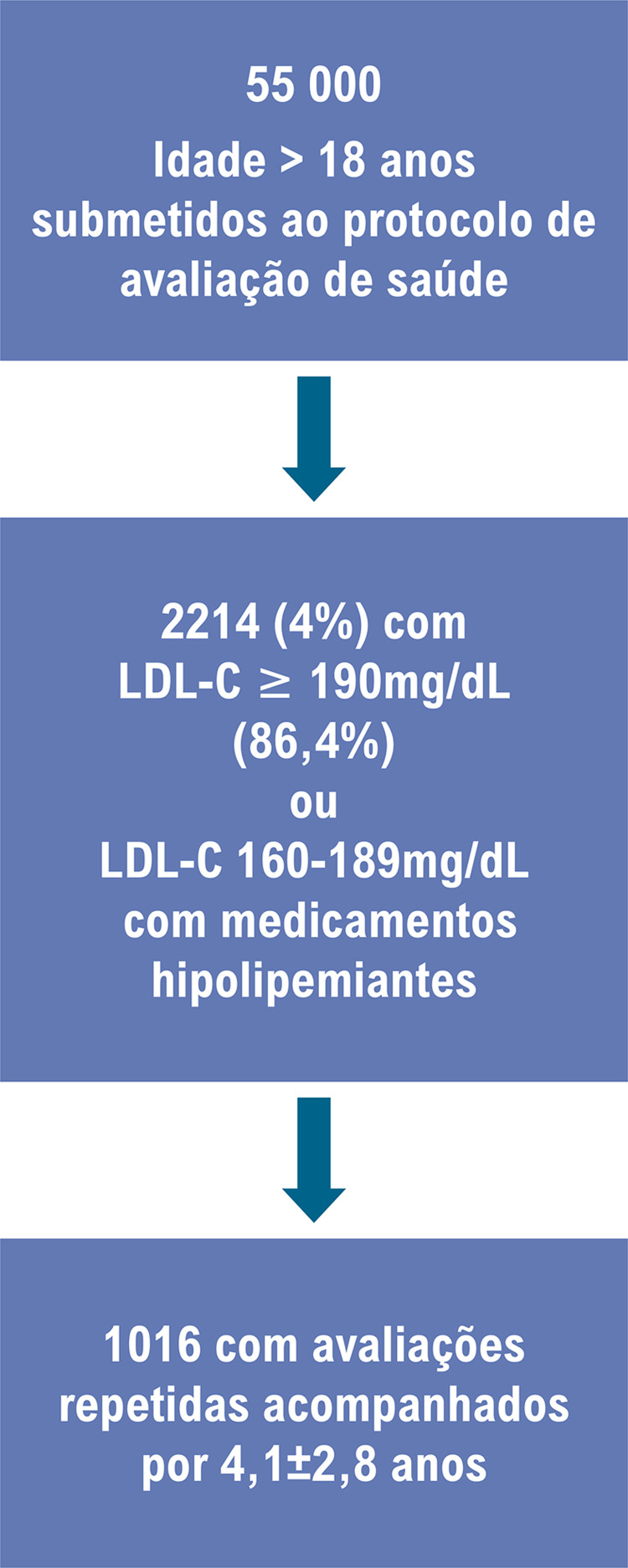
– Fluxograma da seleção e categorização dos pacientes.

A Figura 1 ilustra o fluxograma da seleção e categorização dos pacientes. Um total de 55000 indivíduos foram avaliados, dos quais 2214 (4%) apresentavam HG, e 1016 (45.8%) foram avaliados mais de uma vez. A Tabela 1 apresenta características clínicas e laboratoriais de indivíduos na avaliação basal (n=2214), com 86,4% dos pacientes apresentando níveis de LDL-C ≥ 190 mg/dL e 13,6% 160-189 mg/dL com agentes hipolipemiantes. A maioria dos pacientes era de jovens do sexo masculino; praticamente um a cada dez pacientes apresentava síndrome metabólica ou era fumante, e menos de 1% referiu DAC prévia. Embora 62,5% dos pacientes tenham relatado diagnóstico de dislipidemia, somente 19% estavam usando drogas hipolipemiantes, com 5,9% nos casos com LDL-C ≥ 190 mg/dL.

**Tabela 1:** – Características clínicas e laboratoriais dos indivíduos com hipercolesterolemia grave na avaliação basal (n=2214)

	Total (n=2214)	LDL-C ≥ 190 mg/dL (n=1913)	LDL-C 160-189 mg/dL com agentes hipolipemiantes (n=301)
Idade (anos)	44,9±8,8	44,2±8,6	49,1 ± 8,9
Sexo masculino n (%)	1,864 (84,2)	1,620 (84,7)	244 (81,1)
Hipertensão, n (%)	299 (13,5)	228 (11,9)	71 (23,6)
Tabagismo, n (%)	256 (11,6)	220 (11,5)	36 (12)
Diabetes, n (%)	59 (2,7)	28 (1,5)	31 (10,3)
Síndrome metabólica, n (%)	218 (10,0)	180 (9,6)	38 (13,1)
Dislipidemia relatada n (%)	1383 (62,5)	1132 (59,2)	251 (83,4)
DAC n (%)	21 (0,9)	10 (0,5)	11 (3,7)
Total Colesterol total (mg/dL)	281,0 ± 28,0	286,0 ± 26,0	249,0 ± 17,0
LDL-Colesterol (mg/dL)	203,0 ± 22,0	207,0 ± 19,0	172,0 ± 8,0
HDL-Colesterol (mg/dL)	47,0 ± 11,0	47,0 ± 11,0	47,0 ± 11,0
Triglicerídeos (mg/dL)	148,0 (26,0-1176,0)	148,0 (26,0-1176,0)	143,0 (47,0-671,0)

Valores de triglicerídeos apresentados em mediana e intervalo interquartil; DAC: doença arterial coronariana.

**Figura 2:**
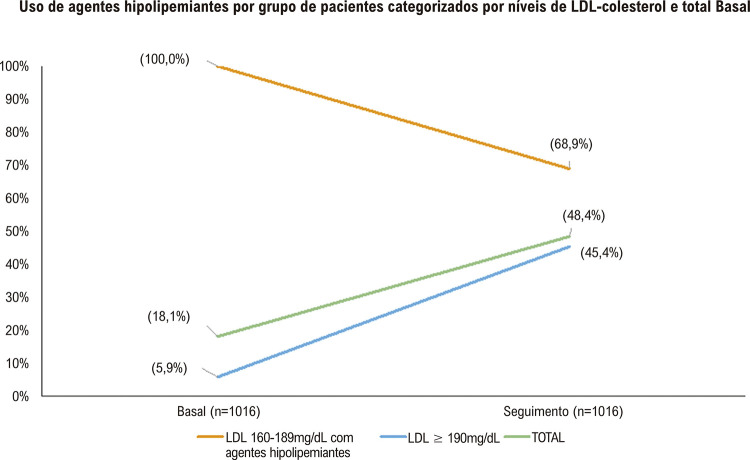
Uso de agentes hipolipemiantes por grupo de pacientes categorizados por níveis de LDL-colesterol; teste do qui-quadrado, p<0,00001 vs. Basal.

A Tabela 2 mostra as características clínicas e laboratoriais dos indivíduos com avaliações repetidas (n=1016). Como o esperado, esses indivíduos apresentaram características similares a do grupo todo. Durante o acompanhamento, somente cinco (0,5%) indivíduos desenvolveram eventos cardiovasculares. A Figura 2 mostra o uso de terapia com medicamentos hipolipemiantes de acordo com os critérios de dislipidemia durante o acompanhamento de 4,1 ± 2,8 anos. O uso desses agentes aumentou 2,7 – 7,7 vezes, respectivamente, na população total e naqueles com LDL-C ≥ 190 mg/dL (teste do qui-quadrado, p<0,00001 vs. basal para ambos). Por outro lado, 31% dos casos com LDL-C de 160-189 mg/dL e terapia prévia com hipolipemiantes interromperam o uso de medicamentos durante o seguimento.

**Tabela 2:** – Características clínicas e laboratoriais dos indivíduos submetidos a avaliações repetidas

	Total (n=1016)	LDL-C ≥ 190 mg/dL (n=884)	LDL-C 160-189 mg/dL com agentes hipolipemiantes no basal (n=132)
Idade (anos)	44,4 ± 8,0	43,8 ± 7,7	48,1 ± 8,4
Sexo masculino n (%)	899 (88,5)	786 (88,9)	113 (85,6)
Hipertensão, n (%)	122 (12,0)	97 (11,0)	25 (18,9)
Tabagismo, n (%)	109 (10,7)	94 (10,7)	15 (11,4)
Diabetes, n (%)	21 (2,1)	12 (1,4)	9 (6,8)
Síndrome metabólica, n (%)	73 (7,3)	61 (7,0)	12 (9,3)
Dislipidemia relatada n (%)	631 (62,1)	526 (59,5)	105 (79,5)
DAC n (%)	5 (0,5)	3 (0,3)	2 (1,5)
Total Colesterol total (mg/dL)	280,0 ± 26,0	284,0 ± 23,0	248,0 ± 16,0
LDL-Colesterol (mg/dL)	202,0 ± 20,0	206,0 ± 17,0	173,0 ± 8,0
HDL-Colesterol (mg/dL)	47,0 ± 10,0	47,0 ± 10,0	47,0 ± 11,0
Triglicerídeos (mg/dL)	147,0 (26,0-1176,0)	148,0 (26,0-1176,0)	133,0 (55,0-412,0)

Valores de triglicerídeos apresentados em mediana e intervalo interquartil; DAC: doença arterial coronariana.

A Tabela 3 apresenta mudanças nos níveis de LDL-C durante o seguimento; no geral, observou-se uma redução média de 26,7% nos níveis de LDL-C (p<0,0001). Reduções ≥ 50% no LDL-C foram alcançadas em 19,2% de todos os indivíduos, 19,1% em indivíduos com LDL ≥ 190 mg/dL e 19,7% daqueles com LDL 160-189 mg/dL em uso de medicamentos hipolipemiantes no basal.

**Tabela 3:** – Mudanças nas concentrações de LDL-colesterol (LDL-C) durante o seguimento

	Total (n=1016)	LDL-C ≥ 190 mg/dL (n=884)	LDL-C 160-189 mg/dL com com agentes hipolipemiantes no basal (n=132)	p
LDL-C no basal (mg/dL)	202,0±20,0	206,0±18,0	173,0±8,0	< 0,0001^1^
LDL-C no seguimento (mg/dL)	146,0±46,0	149,0±46,0	132,0±47,0	<0,0001^1^
Média de mudança (%) no LDL-C	-26,7%	-26,1%	-24%	-

LDL: Lipoproteína de baixa densidade; ^1^ teste t pareado.

**Figura 3:**
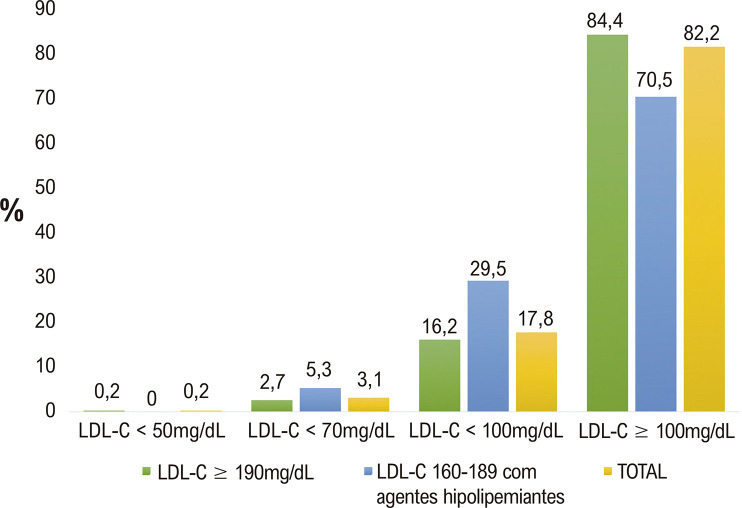
– Porcentagens dos pacientes com hipercolesterolemia grave no basal que atingiram as metas de LDL-colesterol (<100 mg/dL, <70 mg/dL, and < 50 mg/dL).

A Figura 3 mostra as porcentagens de indivíduos que atingiram metas de LDL-C < 100mg/dL, <70 mg/dL, e < 50 mg/dL em todos os pacientes e nos subgrupos com HG. A maioria dos indivíduos não alcançaram as metas recomendadas pelas diretrizes.^3,4^

As Tabelas 4 e 5 mostram associações univariada e multivariada das variáveis clínicas com o uso de terapia medicamentosa hipolipemiante na última visita. Idade mais avançada, diagnóstico prévio de hipertensão ou dislipidemia, um número maior de consultas, e um período mais longo de acompanhamento foram independentemente associados com o uso de terapia hipolipemiante.

**Tabela 4:** – Comparações das variáveis clínicas entre pacientes que usavam medicamentos hipolipemiantes e pacientes que não os usavam na última visita clínica

	Total (n=1016)	Não usavam agentes hipolipemiantes (n=524)	Usavam agentes hipolipemiantes (n=492)	p
Idade (anos)^1^	44,4 ± 8,0	43,1± 7,7	45,7 ± 8,0	< 0,0001
Sexo masculino n (%)^2^	899 (88,5)	464 (88,5)	435 (88,4)	0,9463
DAC n (%)^3^	5 (0,5)	1 (0,2)	4 (0,8)	0,204
Hipertensão n (%)^2^	122 (12)	47 (9)	75 (15,2)	0,0021
Diabetes n (%)^2^	21 (2,1)	10 (1,9)	11 (2,2)	0,714
Dislipidemia n (%)^2^	631 (62,1)	287 (54,8)	344 (69,9)	<0,0001
Tabagismo n (%)^2^	109 (10,7)	60 (11,5)	49 (10)	0,4303
Síndrome metabólica n (%)^2^	73 (7,3)	40 (7,8)	33 (6,8)	0,547
Número de consultas^1^	1,7 ± 1,2	1,6 ± 1,2	1,8 ± 1,3	0,0025
Intervalo entre a primeira e a última consulta (anos)^1^	4,1 ± 2,8	3,5 ± 2,4	4,8 ± 3,0	< 0,0001

DAC: doença arterial coronariana; ^1^Teste t de Student; ^2^teste do qui-quadrado; ^3^Teste exato de Fisher.

**Tabela 5:** – Associações multivariadas das variáveis clínicas com o uso de medicamentos hipolipemiantes na última consulta

Parâmetro	OR (IC95%)	p
Idade (anos)	1,05 (1,03; 1,07)	<0,0001
Diagnóstico de hipertensão	1,72 (1,12; 2,65)	0,0139
Diagnóstico de dislipidemia	2,22 (1,65; 2,99)	<0,0001
Número de consultas	1,21 (1,08; 1,35)	0,0011
Intervalo entre a primeira e a última consulta (anos)	1,15 (1,06; 1,23)	0,0004

Modelo logístico multivariado stepwise; teste Hosmer e Lameshow: p = 0,2547; OR: odds ratio (IC95%: Intervalo de Confiança de 95%).

## Discussão

Foi encontrado um hiato significativo no controle do LDL-C neste grupo de indivíduos com HG e de alto risco para DCVA submetidos a pelo menos uma avaliação de saúde de rotina (Figura Central). Esse hiato persistiu por um período médio de 4,1 anos de acompanhamento em que avaliações repetidas foram realizadas. Vale ressaltar que oito em 10 indivíduos persistiram com concentrações elevadas de LDL-C, e somente 19,2% alcançaram a redução recomendada de pelo menos 50% no LDL-C e 3,1% a meta de LDL-C <70mg/dL. Daqueles com LDL-C ≥ 190 mg/dL, 55% persistiram sem uso de medicamentos hipolipemiantes apesar das claras recomendações de diretrizes sobre seu uso.^2-4^ Ainda, praticamente três em 10 indivíduos com LDL-C 160-189 mg/dL no basal interromperam a terapia farmacológica.

A redução de LDL-C está entre as medidas preventivas mais importantes para mitigar o risco de DCVA. Indivíduos com HS são considerados em risco mais elevado ao longo da vida mesmo sem outros fatores de risco ou manifestações clínicas prévias de aterosclerose.^2-4^ Considerando esse risco, as diretrizes indicam uma redução robusta nos níveis de LDL, isto é, de pelo menos 50% dos níveis basais, e documentos brasileiros e europeus recomendam a manutenção de valores de LDL-C abaixo de 70mg/dL. Para isso, além das mudanças no estilo de vida, recomenda-se terapia farmacológica com altas doses de estatinas de alta potência. Contudo, considerando as altas concentrações de LDL-C nos indivíduos deste estudo, terapias combinadas com ezetimibe, ácido bempedoico ou inibidores da pró-proteína convertase subtilisina quexina tipo 9 (PCSK9), anticorpos monoclonais ou pequenos RNA de interferência (siRNA), pode ser necessária para se atingir as metas de LDL-C. De fato, sugeriu-se recentemente que terapias combinadas reduzem os níveis de colesterol, atingindo-se as metas de LDL-C em indivíduos em risco alto ou muito alto para DCVA.^10^Tal recomendação deriva de evidência robusta de que estatinas, eztimibe e inibidores monoclonais da PCSK9 reduzem DCVA e que tal efeito depende da redução dos níveis de LDL-C.^11,12^

A HG pode indicar a presença de formas genéticas de dislipidemias, como a hipercolesterolemia familiar e a hipercolesterolemia poligênica. Evidência recente indica que um histórico genético comprovado para dislipidemias está associado com risco mais alto de DCVA em comparação a indivíduos em que variantes genéticas não são encontradas e similarmente apresentam níveis elevados de LDL-C. Isso provavelmente ocorre devido à exposição prolongada a níveis muito altos de LDL-C, principalmente nos casos de HG.^13,14^

Anteriormente, em um grupo menor de indivíduos com HG derivada da mesma população de que se originaram, Santos et al.^7^ encontraram, em uma avaliação transversal, baixo conhecimento sobre HG e suas consequências, como início precoce de aterosclerose, necessidade de farmacoterapia para reduzir os níveis de LDL-C, e rastreamento em cascata por parentes assintomáticos.

Evidências dos estudos Da Vinci^15^ e Santorini^16^ com populações europeias contemporâneas indicam que a maioria dos indivíduos com risco alto ou muito alto persistem com concentrações inadequadas de colesterol. Metas de LDL-C mais rígidas, com diretrizes mais recentes propondo valores ainda mais baixos de LDL-C que no passado,^17^ e o baixo uso de terapias combinadas podem justificar esses achados.^15^ O atual estudo EMR mostra que o controle inadequado de LDL-C em indivíduos em alto risco no Brasil não é circunstancial, uma vez que os resultados persistem durante avaliações repetidas. Vale destacar que, no basal, apesar de 59% dos indivíduos com LDL-C ≥190 mg/dL estudados relataram um diagnóstico prévio de dislipidemia, 94% não estavam usando medicamentos hipolipemiantes. Durante o acompanhamento, o uso desses fármacos subiu para 45%, enquanto todos os pacientes deveriam estar em tratamento segundo as diretrizes. De fato, essa é uma das razões por que 81% dos participantes do estudo não atingiram as metas recomendadas de redução ≥50% nos níveis de LDL-C e de 97% deles não mantiveram os níveis de LDL-C abaixo de 70mg/dL. Outro achado importante que pode justificar os resultados no grupo todo e que 31% daqueles com LDL-C 160-189mg/dL em uso de agentes hipolipemiantes no basal interromperam o uso dos medicamentos.

Para modificar os achados negativos deste de outros estudos,^17-19^é essencial identificar possíveis causas para a falta do uso de medicamentos hipolipemiantes. De fato, idade avançada, seguimentos mais longos, um número maior de consultas, e presença prévia de fatores de risco para aterosclerose foram independentemente associados com uso de agentes hipolipemiantes na última consulta do seguimento. Assim, a idade relativamente baixa dos pacientes, a baixa frequência de doença cardiovascular prévia, e de eventos cardiovasculares incidentes durante a acompanhamento, o pequeno número de consultas e a possibilidade de má percepção do risco de DCVA pelos indivíduos estudados podem haver contribuído para o achado. Katz et al.^8^ observaram uma má percepção do alto risco de DCVA em 6544 indivíduos que se submeteram ao mesmo protocolo de avaliação. Quando um período de uma vida foi aplicado em vez do período de 10 anos normalmente utilizado para avaliar o risco de DCVA, 91,2% dos pacientes em alto risco foram considerados indivíduos que percebiam mal o risco. Conceição et al.^6^ relataram um agravamento nos fatores de risco para DCVA, exceto na frequência de tabagismo, em indivíduos submetidos a avaliações médicas repetidas, mostrando, claramente, que a detecção dos fatores de risco não é suficiente para reduzir o risco de DCVA. Também é importante esclarecer que médicos no protocolo de avaliação de saúde usualmente não prescrevem medicamentos hipolipemiantes e, na maioria das situações, dão conselhos sobre resultados e os encaminham para atendimento médico próprio. De fato, tempos mais curtos de acompanhamentos e menor número de visitas no programa de avaliação de saúde foram indicadores independentes de falta de uso de hipolipemiantes.

Este estudo tem várias limitações. Primeiro, os participantes do estudo não representam completamente a população brasileira, considerando o alto nível socioeconômico e a predominância de sexo masculino. Contudo, uma avaliação transversal do estudo ELSA-Brasil, um estudo epidemiológico mais amplo realizado com funcionários públicos de seis grandes áreas urbanas brasileiras, também mostrou que, nos indivíduos considerados de alto risco para doenças cardíacas equivalentes à DAC, somente 38,6% apresentavam concentrações de LDL-C de acordo com metas recomendadas.^18^ Ainda, recentemente, Machline-Carrion et al.^20^ e Santo et al.^21^ encontraram lacunas importantes no tratamento adequado com agentes hipolipemiantes na prevenção secundária^20^ e primária^21^ de pacientes atendidos na atenção primária. Resultados do estudo atual apresentam uma situação preocupante considerando o acompanhamento prospectivo. Segundo, nem todos os indivíduos com HG compareceram em mais de uma visita, mas as características basais daqueles submetidos a uma avaliação não foram diferentes das dos pacientes acompanhados. Terceiro, este relatório retrata um único centro de São Paulo, e pode não representar a realidade de programas similares no país. Finalmente, não foi possível determinar a ocorrência de eventos adversos relacionados à estatina, que poderiam ter levado à não adesão à terapia. Os pontos fortes do estudo são a repetição de um protocolo padronizado durante o acompanhamento e a identificação de fatores de risco para DCVA.

## Conclusões

Neste estudo de EMR, encontrou-se um hiato grave e persistente no controle do LDL-C em indivíduos com HG. A idade relativamente baixa dos participantes, e o pequeno número de consultas, a baixa ocorrência de eventos cardiovasculares, e uma má-percepção do risco pode explicar, em parte, os resultados do estudo. Intervenções mais adequadas, consistindo em um maior número de consultas, fontes de telemedicina, e uma abordagem multiprofissional para aumentar a percepção sobre os benefícios do controle da dislipidemia, podem ser necessárias para a prevenção primária de alto risco, como mostrado para indivíduos com DCVA prévia.^17^
