# Supplementary material for: Colesterol Não Controlado em Indivíduos com Hipercolesterolemia Grave Acompanhados em um Programa de Avaliação da Saúde no Brasil
Source: Arq Bras Cardiol. 2024 Oct 30;121(11):e20240116. [Article in Portuguese] doi: 10.36660/abc.20240116 (PMC11634292; doi:10.36660/abc.20240116)
Supplement: Translation

# Translation: Uncontrolled Cholesterol in Individuals with Severe Hypercholesterolemia in a Health Evaluation Program in Brazil

**DOI:** 10.36660/abc.20240116i

**Authors:** Raul D. Santos, Nea Miwa Kashiwagi, Fernando Yue Cesena, Silvia Regina Lamas Assis, Josué Nieri, Carlos Andre Minanni, Marcelo Franken, Otavio Berwanger

**Affiliations:** 1 Brazil Hospital Israelita Albert Einstein, São Paulo, SP – Brazil; 2 Brazil Instituto do Coração do Hospital das Clínicas da Faculdade de Medicina da Universidade de São Paulo, São Paulo, SP – Brazil; 3 Brazil Instituto Dante Pazzanese de Cardiologia, São Paulo, SP – Brazil; 4 Brazil Cenocor, Guarulhos, SP – Brazil; 5 United Kingdom George Institute for Global Health UK, London – United Kingdom

**Keywords:** Cholesterol, Heart Disease Risk Factors, Hypercholesterolemia

## Abstract

**Background:**

Individuals with severe hypercholesterolemia (SH) are considered at high atherosclerosis risk and should be intensively treated with lipid-lowering drugs aiming for an LDL-C reduction of≥50% and a goal of <70 mg/dL.

**Objectives:**

This study aimed to evaluate cholesterol control in individuals with SH (LDL-C ≥ 190 mg/dL or 160-189 mg/dL using lipid-lowering drugs) followed in a health evaluation program.

**Methods:**

55,000 individuals were evaluated, of which 2,214 (4%) had SH, and 1,016 (45.8%) had repeated assessments. Achievement of recommended LDL-C goals was the primary study endpoint. A p-value < 0.05 was considered significant.

**Results:**

Mean age (± SD) was 44.9±8.8 years, 84.2% were men, and 0.5% reported previous myocardial infarction. Mean LDL-C was 203.0±22.0 mg/dL, and although 62.5% referred dyslipidemia, only 19% were using lipid-lowering drugs (5.9% in cases with LDL-C ≥ 190 mg/dL). During a 4.1±2.8-year follow-up, use of lipid-lowering drugs increased from 18.1% to 48.4% (p<0.00001), 5.9% to 45.4% in those with LDL-C ≥ 190 mg/dL (p< 0.00001) though 31% of cases with LDL-C 160-189 mg/dL stopped taking medications. Overall, there was a mean 26.7% reduction in LDL-C (p<0.0001), and LDL-C reductions ≥50% were attained in 19.2%, 19.1%, and 19.7 % of all individuals, and in those with LDL-C > 190 mg/dL and 160-189 mg/dL respectively. Only 3.1% reached LDL-C < 70 mg/dL (2.7% in those with LDL-C ≥ 190 and 5.3% in those with 160-189 mg/dL).

**Conclusions:**

A serious gap was found between treatment recommendations and reality in individuals at high atherosclerosis risk due to SH.

## Introduction

Atherosclerotic cardiovascular disease (ASCVD) is the leading cause of death in Brazil.^1^ Plasma LDL-cholesterol (LDL-C) is a causal risk factor for ASCVD.^2-4^ The determination of LDL-C concentrations, therefore, is an essential tool for estimating ASCVD risk and implementing preventive therapies. Adults with severe hypercholesterolemia (SH) defined as LDL-C ≥ 190 mg/dL, are classified as at high ASCVD risk independently of other risk conditions or factors, and guidelines recommend pharmacological lipid-lowering therapy in addition to lifestyle changes to prevent the onset of ASCVD. For these individuals, US, Brazilian, and European guidelines recommend LDL-C reductions of at least 50% with LDL-C goals < 70 mg/dL in the latter two guidelines.^2-4^ In addition, persistent LDL-C concentrations between 160 and 189 mg/dL are associated with an elevated lifetime risk of ASCVD, are considered risk enhancers and favor the initiation of statin therapy.^2,5^

Health evaluation or checkup programs aim to identify risk factors for ASCVD and refer individuals to adequate medical care when necessary.^6^ Previously, we detected an important gap in ASCVD risk perception, cholesterol management, and familial hypercholesterolemia (FH) awareness in individuals with SH submitted to a routine health evaluation.^7^ These findings complemented another observation showing an inadequate overall ASCVD risk perception by individuals at risk that might have deleterious consequences for controlling risk factors and preventing ASCVD.^8^ The lack of adequate control of risk factors or disease states and absence of compliance with recommendations after checkup programs is a challenge to preventive medicine programs^6^ and needs to be addressed. The objective of this study was to generate real-world evidence (RWE) on the efficacy of LDL-C control according to recent guidelines in individuals with SH submitted to repeated evaluations in a routine health evaluation program. The primary study endpoint was attaining recommended LDL-C goals (reduction ≥50% and/or LDL-C <70 mg/dL) for these high-risk individuals during follow-up.

## Methods

This is a retrospective evaluation of prospectively collected data from adults undergoing a routine health evaluation protocol at an outpatient clinic of a tertiary hospital in the city of Sao Paulo, Brazil. Most participants worked in companies that offered routine health evaluations to their employees. This study was approved by the ethics committee (CAAE number 25772619.0000.00.0071), and a waiver for informed consent was obtained. This study was funded by an unrestricted grant from Amgen Laboratories Brazil, and the funding source had no role in data collection, analysis, interpretation, study conclusions, and manuscript writing.

The inclusion criteria were a) individuals aged ≥ 18 years with SH defined as an LDL-C ≥190mg/dL or b) LDL-C between 160 and 189 mg/dL in those using pharmacological lipid-lowering therapy followed at the outpatient clinic between October 2004 and November 2019. The health evaluation protocol consisted of clinical examination (use of standard questionnaires for previous ASCVD and its risk factors, blood pressure, diabetes, dyslipidemia, and medications for their control), physical examination, and fasting laboratory tests as previously described.^7,9^ An evaluation report was sent to either participants or company occupational physicians when available, and participants were invited to a second interview with examining physicians to discuss test results. Participants were advised and referred to a specific disease specialist in case of abnormal test results. Usually, no medications were prescribed by the check-up physician. The following parameters were extracted from electronic medical records: age, sex, body mass index, presence of ASCVD risk factors such as arterial hypertension, diabetes, dyslipidemia, current smoking, family history of early coronary heart disease (CHD) (<55 and < 65 years old respectively in male and female first-degree relatives), metabolic syndrome defined according to the International Diabetes Federation,^4^ and previous CHD defined as myocardial infarction, angina or revascularization. Data on fasting lipid profiles (total and LDL-C, HDL-cholesterol, and triglycerides) were also collected.

In subjects evaluated on more than one occasion, information was collected regarding the use or not of lipid-lowering drugs and the achievement of LDL-C goals according to guideline recommendations, i.e., percentage of individuals with LDL-C <100 mg/dL, <70 mg/dL, and <50 mg/dL in the follow-up.^3,4^ The mean percentage changes in LDL-C from the first evaluation and the percentage of those attaining LDL-C lowering ≥ 50% from baseline as recommended by guidelines^2-4^ for high ASCVD risk subjects were also determined. Data from the last visit were analyzed for those with more than one follow-up evaluation.

### Statistical analysis

Data normality was tested by the Kolmogorov-Smirnov test; continuous data with a Gaussian distribution are shown as mean standard deviation (SD), non-Gaussian data are shown as medians (interquartile ranges), and categorical variables are shown as absolute and relative frequencies.

For the analysis, study subjects were initially categorized and grouped according to LDL-C (mg/dL) values during follow-up as > 100, 100-70, <70-50, and <50. Descriptive statistics were performed, and groups were compared by Student’s paired t-test. Categorical data were compared using the chi-squared test or Fisher’s exact test. Univariate and multivariate stepwise logistic regression models were used to evaluate the association of clinical variables of interest with pharmacological lipid-lowering therapy at the last clinical evaluation during follow-up. A two-tailed p-value < 0.05 was considered significant. All analyses were performed using the R software version 4.1.1 (R Foundation).

## Results

Figure 1 illustrates the flowchart of patient selection and categorization. A total of 55,000 individuals were evaluated, of which 2,214 (4%) had SH, and 1,016 (45.8%) were assessed more than once. Table 1 shows clinical and laboratory characteristics of individuals at baseline evaluation (n=2,214), with 86.4% of patients presenting LDL-C ≥ 190 mg/dL and 13.6% presenting 160-189 mg/dL with lipid-lowering drugs. This is a young, predominantly male population; roughly one in ten had metabolic syndrome or were smokers, and less than 1% referred previous CHD. Although 62.5% reported previous dyslipidemia diagnosis, only 19% were using lipid-lowering drugs, with 5.9% in cases with LDL-C ≥ 190 mg/dL.

**Figure 1:** – Flowchart of patient selection and categorization; LLT: pharmacological lipid-lowering therapy.

**Table 1:** – Clinical and laboratory characteristics of individuals presenting severe hypercholesterolemia at baseline evaluation (n=2,214)

	Total (n=2,214)	LDL-C ≥ 190 mg/dL (n=1,913)	LDL-C 160-189 mg/dL with lipid-lowering medications (n=301)
Age (years)	44.9±8.8	44.2±8.6	49.1 ± 8.9
Male sex, n (%)	1.864 (84.2)	1.620 (84.7)	244 (81.1)
Hypertension, n (%)	299 (13.5)	228 (11.9)	71 (23.6)
Smoking, n (%)	256 (11.6)	220 (11.5)	36 (12)
Diabetes, n (%)	59 (2.7)	28 (1.5)	31 (10.3)
Metabolic syndrome, n (%)	218 (10.0)	180 (9.6)	38 (13.1)
Referred Dyslipidemia, n(%)	1383 (62.5)	1132 (59.2)	251 (83.4)
CHD n (%)	21 (0.9)	10 (0.5)	11 (3.7)
Total Cholesterol (mg/dL)	281.0 ± 28.0	286.0 ± 26.0	249.0 ± 17.0
LDL-Cholesterol (mg/dL)	203.0 ± 22.0	207.0 ± 19.0	172.0 ± 8.0
HDL-Cholesterol (mg/dL)	47.0 ± 11.0	47.0 ± 11.0	47.0 ± 11.0
Triglycerides (mg/dL); median (ranges)	148.0 (26.0-1176.0)	148.0 (26.0-1176.0)	143.0 (47.0-671.0)

CHD: coronary heart disease.

Table 2 shows the clinical and laboratory characteristics of individuals with repeated evaluations (n=1,016). As expected, these individuals had characteristics similar to those of the whole group. During follow-up, only 5 (0.5%) individuals developed CHD events. Figure 2 shows the use of lipid-lowering therapy according to dyslipidemia criteria during the 4.1 ± 2.8-year follow-up. The use of lipid-lowering drugs increased 2.7-fold and 7.7-fold, respectively, in the whole population and those with LDL-C ≥ 190 mg/dL (Chi^2^ test, p< 0.00001 vs. baseline for both). On the other hand, 31% of cases with LDL-C 160-189 mg/dL and previous lipid-lowering therapy stopped taking medications at follow-up.

**Table 2:** – Clinical and laboratory characteristics of individuals undergoing repeated evaluations

	Total (n=1,016)	LDL-C ≥ 190 mg/dL (n=884)	LDL-C 160-189 mg/dL with lipid-lowering medications at baseline (n=132)
Age (years)	44.4 ± 8.0	43.8 ± 7.7	48.1 ± 8.4
Male sex n (%)	899 (88.5)	786 (88.9)	113 (85.6)
Hypertension n (%)	122 (12.0)	97 (11.0)	25 (18.9)
Smoking n (%)	109 (10.7)	94 (10.7)	15 (11.4)
Diabetes n (%)	21 (2.1)	12 (1.4)	9 (6.8)
Metabolic syndrome n (%)	73 (7.3)	61 (7.0)	12 (9.3)
Dyslipidemia n (%)	631 (62.1)	526 (59.5)	105 (79.5)
CHD n (%)	5 (0.5)	3 (0.3)	2 (1.5)
Total Cholesterol	280.0 ± 26.0	284.0 ± 23.0	248.0 ± 16.0
LDL-Cholesterol	202.0 ± 20.0	206.0 ± 17.0	173.0 ± 8.0
HDL-Cholesterol	47.0 ± 10.0	47.0 ± 10.0	47.0 ± 11.0
Triglycerides	147.0 (26.0-1176.0)	148.0 (26.0-1176.0)	133.0 (55.0-412.0)

Lipids in mg/dL; triglyceride values are shown as median (ranges); CHD: coronary heart disease.

**Figure 2:** – Use of lipid-lowering therapy by LDL-cholesterol (LDL-C) level groups; chi-squared 2 test, p<0.00001 vs. baseline.

Table 3 shows changes in LDL-C during follow-up; overall, there was a mean 26.7% reduction in LDL-C (p<0.0001). LDL-C reductions ≥50% were attained in 19.2%, 19.1%, and 19.7 %, respectively, in all individuals, in those with LDL-C ≥ 190 mg/dL and those with 160-189 mg/dL with lipid-lowering medications at baseline.

**Table 3:** – Changes in LDL-cholesterol levels during follow-up

	Total (n=1,016)	LDL-C ≥ 190 mg/dL (n=884)	LDL-C 160-189 mg/dL with lipid lowering medications at baseline (n=132)	p
LDL-C at baseline (mg/dL)	202.0±20.0	206.0±18.0	173.0±8.0	< 0.0001^1^
LDL-C at follow-up (mg/dL)	146.0±46.0	149.0±46.0	132.0±47.0	<0.0001^1^
Mean % change in LDL-C	-26.7%	-26.1%	-24%	-

LDL: low-density lipoprotein; ^1^ paired t-test.

Figure 3 shows the percentages of SH individuals achieving LDL-C goals (<100 mg/dL, <70 mg/dL, and < 50 mg/dL). Most individuals did not achieve recommended goals according to guidelines recommendations.^3,4^

**Figure 3:** – Percentages of individuals with severe hypercholesterolemia at baseline achieving LDL-cholesterol goals (<100 mg/dL, <70 mg/dL, and < 50 mg/dL).

Tables 4 and 5 show univariate and multivariate associations of clinical variables with the use of pharmacological lipid-lowering therapy on the last visit. Older age, previous diagnosis of hypertension or dyslipidemia, a higher number of medical visits, and a longer follow-up duration were independently associated with the use of lipid-lowering therapy.

**Table 4:** – Comparisons of clinical variables in those using or not lipid-lowering medications on the last medical visit

	Total (n=1016)	Not in use of lipid lowering medications (n=524)	In use of lipid lowering medications (n=492)	p
Age (years)^1^	44.4 ± 8.0	43.1± 7.7	45.7 ± 8.0	< 0.0001
Male sex n (%)^2^	899 (88.5)	464 (88.5)	435 (88.4)	0.9463
CHD n (%)^3^	5 (0.5)	1 (0.2)	4 (0.8)	0.204
Hypertension n (%)^2^	122 (12)	47 (9)	75 (15.2)	0.0021
Diabetes n (%)^2^	21 (2.1)	10 (1.9)	11 (2.2)	0.714
Dyslipidemia n (%)^2^	631 (62.1)	287 (54.8)	344 (69.9)	<0.0001
Smoking n (%)^2^	109 (10.7)	60 (11.5)	49 (10)	0.4303
Metabolic syndrome n (%)^2^	73 (7.3)	40 (7.8)	33 (6.8)	0.547
Number of medical visits^1^	1.7 ± 1.2	1.6 ± 1.2	1.8 ± 1.3	0.0025
Time interval between first and last visits (years)^1^	4.1 ± 2.8	3.5 ± 2.4	4.8 ± 3.0	< 0.0001

CHD: coronary heart disease; ^1^: Student’s t test; ^2^: Chi^2^ test ; ^3^: Fisher’s exact test.

**Table 5:** – Multivariate associations of clinical variables with the use of lipid-lowering medications on the last medical visit

Parameter	OR (95%CI)	p
Age (years)	1.05 (1.03; 1.07)	<0.0001
Hypertension diagnosis	1.72 (1.12; 2.65)	0.0139
Dyslipidemia diagnosis	2.22 (1.65; 2.99)	<0.0001
Number of consultations	1.21 (1.08; 1.35)	0.0011
Time interval between first and last visits (years)	1.15 (1.06; 1.23)	0.0004

Multivariate stepwise logistic model; Hosmer and Lameshow test: p= 0.2547; OR: odds ratio (95% CI: confidence interval).

## Discussion

A significant hiatus in LDL-C control was encountered in this group of individuals with SH and considered to be at high ASCVD risk who were submitted to at least one routine health evaluation (Central Illustration). This hiatus persisted during a mean 4.1-year follow-up where repetitive assessments were performed. Of importance, 8 in 10 individuals persisted with elevated LDL-C concentrations, and only 19.2% and 3.1% attained the recommended LDL-C reduction of at least 50% and an LDL-C goal of < 70 mg/dL, respectively. Of those with LDL-C ≥ 190 mg/dL, 55% persisted without lipid-lowering pharmacological therapies despite the clear recommendations of guidelines for their use.^2-4^ Furthermore, roughly 3 in 10 individuals with LDL-C 160-189 mg/dL at baseline stopped pharmacological therapy.

Reduction of LDL-C is among the most important preventive measures to mitigate the risk of ASCVD. Those with SH are considered to have a high lifetime risk even without other risk factors or previous clinical manifestations of atherosclerosis.^2-4^ Considering this risk, guidelines indicate robust LDL-C lowering, i.e., at least 50% from baseline levels, and Brazilian and European documents recommend attaining an LDL-C value < 70 mg/dL. For that, in addition to changes in lifestyle, pharmacological therapy with high-dose, high-potency statins is recommended. However, considering the very high LDL-C in these individuals seen in this study, association therapies like ezetimibe, bempedoic acid, or proprotein convertase subtilisin kexin type 9 (PCSK9) inhibitors, either monoclonal antibodies or small interfering RNA drugs, may be necessary to attain the LDL-C goals. Indeed, combination therapies have been recently suggested to reduce cholesterol and achieve the proposed LDL-C goals in individuals at high and very high risk for ASCVD.^10^ This recommendation derives from robust evidence that statins, ezetimibe, and monoclonal PCSK9 inhibitors reduce ASCVD and that this effect depends on LDL-C lowering.^11,12^

SH may indicate the presence of genetic forms of dyslipidemias like familial or polygenic hypercholesterolemia. Recent evidence indicates that a proven genetic background for dyslipidemias is associated with higher ASCVD risk compared to individuals where genetic variants are not encountered, yet they have similar high LDL-C concentrations. This probably occurs due to prolonged exposure to very high LDL-C, mainly in the case of FH.^13,14^

Previously, in a smaller group of individuals with SH derived from the same population where the participants of this study originated, Santos et al.^7^ found, in a cross-sectional evaluation, low awareness of either FH or its consequences, like early atherosclerosis onset, the need for pharmacological therapy to reduce LDL-C, and cascade screening of asymptomatic relatives.

Evidence from the DaVinci^15^ and Santorini^16^ studies in contemporary European populations indicate that most high and very high-risk individuals persist with inadequate cholesterol concentrations. Stricter LDL-C goals, with recent guidelines proposing even lower LDL-C than in the past,^17^ and the low use of combination therapies may justify these findings.^15^ The current RWE study shows that inadequate control of LDL-C in high-risk Brazilian individuals is not circumstantial since findings persist during repeated evaluations. Of importance, at baseline, although 59% of studied individuals with LDL-C ≥190mg/dL reported previous dyslipidemia diagnosis, 94% were not taking lipid-lowering drugs. During follow-up, the use of these medications rose to 45%, while all were supposed to be in use of medications according to guidelines. Indeed, this is one of the reasons why 81% of study participants did not attain the recommended goals of reducing LDL-C ≥ 50% and 97% did no attain LDL-C < 70 mg/dL. Another important finding that may justify the results in the whole group is that 31% of those with LDL-C 160-189 mg/dL who were using lipid-lowering therapies at baseline stopped taking medications.

To change the negative findings of this and other studies,^17-19^ it is essential to identify possible causes for the lack of use of lipid-lowering medications. Indeed, older age, longer follow-ups, a higher number of visits, and the previous presence of risk factors for atherosclerosis were independently associated with pharmacological lipid-lowering therapy use at the last follow-up visit. Thus, the relatively young age of the population, the low frequency of previous cardiovascular disease and incident CHD events during follow-up, the small number of visits, and the possible ASCVD risk misperception by the studied individuals may have contributed to this finding. Previously, Katz et al.^8^ have encountered a misperception of high ASCVD risk in 6,544 individuals who underwent same health evaluation protocol. When a lifetime instead of the usual 10-year estimation was used to evaluate ASCVD risk, 91.2% of high-risk subjects were considered hypo-perceivers. Conceição et al.^6^ have previously reported a worsening of risk factors for ASCVD except for smoking frequency in individuals who underwent repeated medical evaluations, clearly showing that detection of risk factors is not enough to reduce the risk of ASCVD. It is also important to clarify that physicians at the health evaluation protocol usually do not prescribe lipid-lowering medications and, in most situations, only give advice about results and refer them to proper medical care. It is uncertain if participants followed these recommendations. Indeed, shorter follow-ups and fewer medical visits at the health evaluation program were independent indicators of a lack of use of pharmacological lipid-lowering therapy.

This study has several limitations. First, the study participants do not fully represent the Brazilian population, considering the high socioeconomic level and male sex predominance. However, a cross-sectional evaluation of the ELSA-Brasil study, a broader epidemiological study performed with civil servants of six large Brazilian urban areas, also showed that in those considered as high-risk for CHD equivalent, only 38.6% had their LDL-C concentrations according to recommended goals.^18^ Moreover, recently, Machline-Carrion et al.^20^ and Santo et al.^21^ have found important gaps in the use of adequate lipid-lowering therapies in secondary^20^ and primary prevention^21^ individuals seen in primary care. Results of the current study show a worrisome situation considering the prospective follow-up. Second, not all individuals with SH attended more than one visit, but the baseline characteristics of those with one single evaluation and those who were followed are similar. Third, this report was about a single center in Sao Paulo, and it may not represent the reality of similar programs in Brazil. Finally, it was not possible to determine the occurrence of statin-related adverse events that could have led to non-compliance with therapy. The study’s strengths are the repetition of a standardized protocol during follow-up and the ascertainment of risk factors for ASCVD.

## Conclusions

In this RWE study, a severe and persistent hiatus in controlling LDL-C in individuals with SH was found. The relatively young age of study participants, the low number of visits, the small occurrence of CHD events, and risk misperception may partly explain the study findings. More adequate interventions, consisting of more medical visits, telemedicine resources, and a multi-professional approach to increase awareness about the benefits of dyslipidemia control, may be necessary for high-risk primary prevention, as shown for people with previous ASCVD.^17^
